# Supplementary material for: Can Artificial Intelligence Improve the Appropriate Use and Decrease the Misuse of REBOA?
Source: Bioengineering (Basel). 2025 Sep 25;12(10):1025. doi: 10.3390/bioengineering12101025 (PMC12561464; doi:10.3390/bioengineering12101025)
Supplement: Supplementary file 1 [file bioengineering-12-01025-s001.zip › bioengineering-3687458-supplementary.pdf]

bioengineering

MDPI

Table S1. Injuries and corresponding ICD-10 codes.

Injury	ICD-10 Code
Pelvic fracture	S32.82 S32.82XA S32.82XB S32.89XA S32.9XXA S32.8 S32.810B S32.81 S32.811B S32.9XXB S32.810 S32.89 S32.9 S32.810A S32.811A S32.89XB S32.811
Femur fracture	S72.362C S72.441B S72.352B S72.432C S72.141B S72.142C S72.042B S72.131B S72.023C S72.322B S72.122C S72.462C S72.411B S72.041B S72.23XC S72.21XC S72.343C S72.422C S72.423B S72.111B S72.022C S72.463C S72.032C S72.033C S72.332B S72.333B S72.413C S72.353B S72.432B S72.141C S72.042C S72.043C S72.342C S72.032B S72.033B S72.323B S72.21XB S72.062C S72.143C S72.343B S72.422B S72.421B S72.023B S72.021C S72.453C S72.452C S72.463B S72.331B S72.121B S72.461B S72.363B S72.362B S72.442C S72.143B S72.353C S72.441C S72.433B S72.431B S72.132C S72.113C S72.321C S72.322C S72.112C S72.451B S72.123B S72.123C S72.121C S72.031C S72.462B S72.461C S72.443B S72.443C S72.22XC S72.23XB S72.361C S72.442B S72.063B S72.063C S72.351C S72.133B S72.341B S72.341C S72.132B S72.111C S72.021B S72.112B S72.331C S72.412C S72.122B S72.413B S72.323C S72.411C S72.363C S72.361B S72.22XB S72.351B S72.352C S72.433C S72.133C S72.043B S72.421C S72.113B S72.453B S72.332C S72.333C S72.031B S72.412B S72.041C S72.061B S72.061C S72.062B S72.142B S72.431C S72.131C S72.342B S72.423C S72.321B S72.022B S72.451C S72.452B
Hemothorax	S27.1XXA J94.2 S27.1
Pneumothorax	J93.83 S27.0XXS J93.9 S27.0 S27.2 J93 S27.0XXA S27.2XXA J93.8
Thoracic aorta injury	S25.01XA S25.09XA S25.00 S25.00XA I71.01 S25.0 S25.01 S25.02 S25.09 S25.02XA

Table S2. Procedures and corresponding ICD-10 codes.

Procedure	ICD-10 Code
Resuscitative endovascular balloon occlusion of the aorta (REBOA)	04L03DZ 04L03DJ 04L04DZ 02LW3DJ 04L04ZZ
Preperitoneal pelvic packing (PPP)	2W43X5Z 2W03X6Z 2W13X6Z 2W53X5Z 2W53X6Z
External fixation of pelvis (EF)	0QH305Z 0QH235Z 0QH345Z 0QH335Z 0QH205Z 0QH245Z
Chest tube placement	0W9B00Z 0W9B0ZZ 0W9900Z 0W990ZZ
Transfusion RBC	30240P1 30233N1 30230N1 30243N1 30243P1 30233P1 30230P1 30240N1
Transfusion whole blood	30243H1 30230H1 30233H1 30240H1
